# Effect of Nanocrystalline Hydroxyapatite Socket Preservation on Orthodontically Induced Inflammatory Root Resorption

**DOI:** 10.22074/cellj.2015.496

**Published:** 2015-01-13

**Authors:** Massoud Seifi, Ali Arayesh, Nafise Shamloo, Roya Hamedi

**Affiliations:** 1 Department of Orthodontic, Dentofacial Deformities Research Center, Research Institute of Dental Sciences of Shahid Beheshti University of Medical Sciences, Tehran, Iran; 2 School of Dentistry, Shahid Beheshti University of Medical Sciences, Tehran, Iran; 3 Department of Oral and Maxillofacial Pathology, Dental School of Shahid Beheshti University of Medical Sciences, Tehran, Iran; 4 Department of Orthodontic, Dentofacial Deformity Research Center, School of Dentistry, Shahid Beheshti University of Medical Sciences, Tehran, Iran

**Keywords:** Hydroxyapatites, Nanoparticles, Orthodontic, Root Resorption, Tooth Movement

## Abstract

**Objective:**

Orthodontically induced inflammatory root resorption (OIIRR) is considered to be an important sequel associated with orthodontic tooth movement (OTM). OTM after Socket preservation enhances the periodontal condition before orthodontic space closure. The purpose of this study is to investigate the histologic effects of NanoBone®, a new highly nonsintered porous nano-crystalline hydroxyapatite bone on root resorption following OTM.

**Materials and Methods:**

This experimental study was conducted on four male dogs. In each dog, four defects were created at the mesial aspects of the maxillary and mandibular first premolars. The defects were filled with NanoBone®. We used the NiTi closed coil for mesial movement of the first premolar tooth. When the experimental teeth moved approximately halfway into the defects, after two months, the animals were sacrificed and we harvested the area of interest. The first premolar root and adjacent tissues were histologically evaluated. The three-way ANOVA statistical test was used for comparison.

**Results:**

The mean root resorption in the synthetic bone substitute group was 22.87 ± 11.25×10^-4^mm^2^ in the maxilla and 21.41 ± 11.25×10^-4^mm^2^ in the mandible. Statistically, there was no significant difference compared to the control group (p>0.05).

**Conclusion:**

The use of a substitution graft in the nano particle has some positive effects in accessing healthy periodontal tissue following orthodontic procedures without significant influence on root resorption (RR). Histological evaluation in the present study showed osteoblastic activity and remodeling environment of nanoparticles in NanoBone®.

## Introduction

Orthodontically induced inflammatory root resorption (OIIRR) is considered to be an important sequel associated with orthodontic tooth movement (OTM) (1). In approximately 5% of patients who undergo orthodontic treatment, up to 5 mm of tooth root loss can occur (2). However a total of 7-13% of individuals who have not had orthodontic treatment show 1-3 mm of external apical root resorption (RR) on radiograph images (3). Histological RR usually presents as microscopic areas of resorption lacunae on root surfaces. Seventy-five percent of these areas become completely repaired with secondary cellular cementum (4). For a short amount of time, orthodontic force applied to teeth can produce resorption lacuna in the absence of radiographically visible external apical RR (5). Researchers believe that the type of tooth movement from the standpoint of biomechanics such as controlled/uncontrolled tipping or bodily movements, the amount of OTM and presence of cellular/ acellular cementum can influence the amount of OIIRR (6, 7). In extraction cases, more OTM can be predicted and make the adjacent teeth more liable to trauma, cell injury reactions or RR (8).

Socket preservation after orthodontic tooth extraction has been proposed by Seifi and Ghoraishian (9). The aim of socket preservation is limiting the alveolar bone resorption following extraction of teeth for orthodontic tooth movement (10). Three-dimensional alveolar bone resorption may occur following extraction and can be prevented by socket preservation (9, 11). OTM can be immediately initiated following socket preservation without waiting for healing of the recipient site (10). Enhanced rate of OTM, decrease the chance of dehiscence and the reduction of RR are some advantages of socket preservation (11, 12).

Currently, due to autogenic bone graft limitations, use of bone replacement materials has gained attention in all surgical areas (13). Bone graft is extremely effective in orthopedic surgery because this method has several applications in all related subfields and in different anatomic areas (14). Bone source can be autograft from the patient or allograft that comes from other individuals (15). However, there are serious complications that occur in the bone donor site of the autograft technique or risks of disease transmission, infections and immunological reactions caused by a foreign tissue in the allograft technique which have motivated researchers to create new combinations and use substitute synthetic materials to prevent these problems (16-19).

In this regard, numerous studies have been carried out on the use of different combinations in order to reduce the occurrence of resorption and inflammation in the jaw and around dental roots during different periodontal treatments (20). Dental material widely used in craniofacial bone surgeries, such as bio-ceramics that contain calcium phosphate, hydroxyapatite or tricalcium phosphate components have shown interesting and promising results (21-23).

However, due to high temperature sintering during processing, there may be a decrease in material porosity and increased density (24). These factors negatively influence osteoconductivity and resorption at the implantation site (25). These bioceramics may therefore have a longer degradation time and even induce chronic inflammatory processes (26). NanoBone® is a new granular graft material formed by nanocrystalline hydroxyapatite (NHA) components in silica gel matrix. Its application in bone surgeries have multiple advantages (27, 28). The internal surface of NanoBone® is very wide (approximately 84 m^2^/g) due to the existence of basic group of SiOH or SiO in Poly Silicic Acid. So, the dimensions of the porosities contained in silica gel, are from 15 to 25 nanometer, which enhances the materials porosities up to 60% (29). The silica gel stimulates the formation of collagen and bone (30). Indications of NanoBone® includes in us lift and/or sinus floor elevation (open/closed) (31, 32), augmentation of alveolar ridge defects, filling of alveolar cavities for stabilizing the bony alveolar ridge (socket preservation) (33), and alveolar ridge reconstruction (34). Animal experiments that have used NHA in a mini-pig critical size defect model showed a significantly higher rate of bone formation compared to other HA and TCP materials or gelatin sponges. The nearly complete resorption eight months after implantation gave an initial insight into the cellular processes of osteoconduction and early remodeling *in vivo* (35, 36). The recruitment and occurrence of Runx-2-positive osteoblast precursor cells and upregulation of BMP-2 in sites grafted by the NHA in humans has suggested that this material has osteoinductive properties (37). In addition, NHA had a major role in preservation of the alveolar ridge after tooth extraction and could have positive effects on improvement of wounds and prevention of bone atrophy (38, 39).

The majority of studies mentioned showed a main effect on socket bone preservation and dental RR. In addition, extensive efforts have been undertaken to find a way to prevent RR. Hence, the present study was designed with the aim to determine the effect of NanoBone® in reduction or prevention of RR during orthodontic treatments and the amount of OTM as well as the histopathology and morphologic evaluation of these processes.

## Materials and Methods

### Ethical considerations

All animal handling and surgical procedures were approved by The Local Committee for Experimental Animal Research Ethics and conducted according to the Institutional Review Board (IRB) guidelines for the use and care of laboratory animals. This study was approved by the Ethics Committee of the Dental Research Center at Shaheed Beheshti University of Medical Sciences.

### Animal experiments

**Fig 1:**
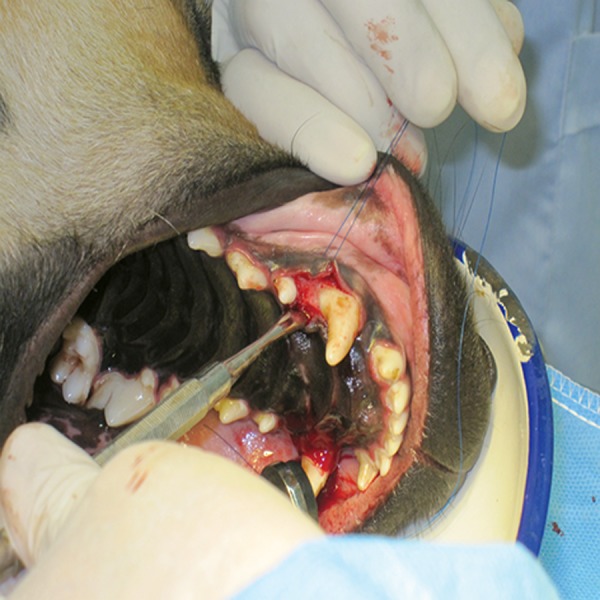
A full-thickness flap from the canine to the first premolar was retracted.

**Fig 2:**
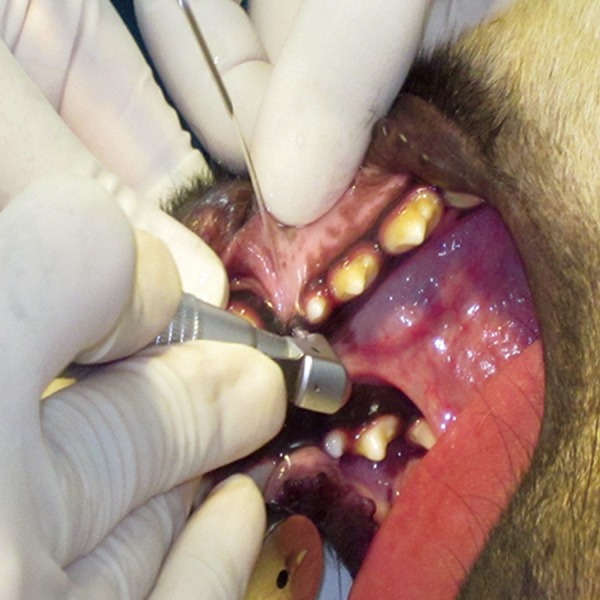
The primary penetration was performed using an implant drill at the mesial side of the lower first premolar in conjunction with cooled saline irrigation.

**Fig 3:**
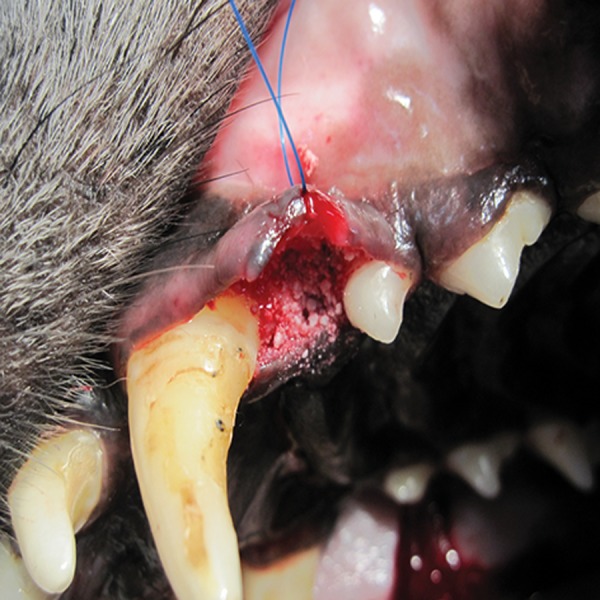
The artificial sockets were filled with NanoBone®.

This research was an experimental, split mouth study. Data were collected by histopathological observations and evaluation of the amount of RR. Samples included 16 quadrants (upper and lower jaws from both right and left sides) in four mixed race male dogs, two years of age that weighed approximately 25 Kg. Method of selection was simple random sampling; the samples were all healthy and each had adequate healthy periodontium. The first premolar and canine were also intact in all dogs. Prior to the onset of the practical part of the study, the animals were maintained under the same conditions for two months at a veterinary clinic for domestic animals where they received vaccinations. For surgery, dogs were anesthetized by 5 mg/kg of 10% ketamine (Parke-Davis, Detroit, MI, USA) administered as an IV. Once anesthetized, dogs’ mouths were completely rinsed with normal saline and chlorhexidine solutions. After injection of local anesthesia, (lidocaine that contained epinephrine), a full-thickness flap from the canine to first premolar was retracted (Fig 1). Then, the primary penetration was performed and we used an implant drill with a 4.3 mm diameter (Nobel Biocare, Yorba Linda, CA, USA) and 10 mm length, to prepare a hole at the mesial side of each first premolar from each quadrant of the animals' jaws (Fig 2). NanoBone® (Artoss, Rostock, Germany) was mixed with normal saline. We prepared the artificial sockets as a standard preparation instead of the extraction sockets and filled them with NanoBone® in the experimental group (8 quadrants) (Fig 3). In the control group (8 quadrants), the artificial sockets had nointervention and were allowed to undergo a normal healing process.

Wound closure was performed by 3/0 nylonsutures (SUPA Medical Devices Co., Tehran, Iran) which remained in the site for ten days.

### Appliance design

**Fig 4:**
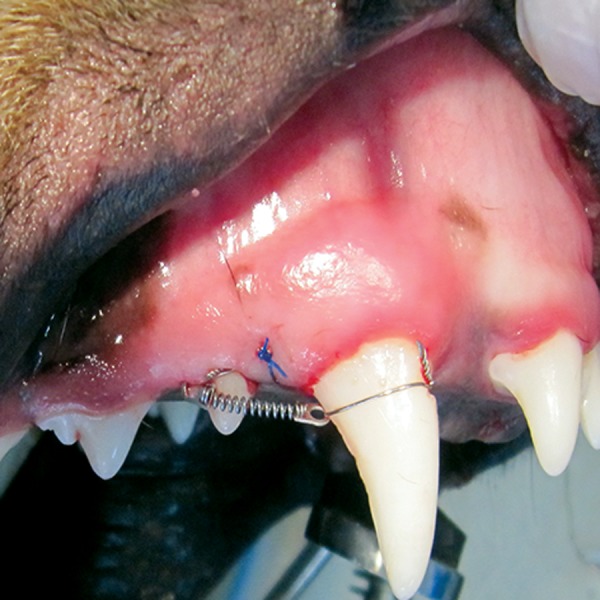
Experimental appliance. An active coiled spring exerted a force of approximately 150 g in the mesial direction.

The first premolar was moved to the mesial side by the application of a 150 g force as measured by a force measuring device, using an NiTi close coil spring that was 9 mm in length (Ormco, Orang, County, CA, USA, Fig 4).

For better mechanical retention of the anchoring ligature wire, we prepared a special fissure in the mesio-gingival section of the canine’s crown where the NiTi coil was fixed with ligature wire (Fig 4). In the disto-gingival part of the first premolar crown the same type of fissure or slot was prepared. Following extension of the NiTi coil, it was tied. By using light-cure composite, wires were stabilized in place. The distance between the first premolar and canine was measured by a digital caliper (Cen-Tech) with a precision of 0.001 inches. This was repeated every two weeks for an eight-week period. During the experiment, dogs’ alimentation was soft (Friskies). In order to prevent infection, dogs received a total of 22 mg/kg of cefazolin (Genian Darou, Iran) administered as IM injections every 8 hours for 3 days. In order to reduce post-surgery pain the animals received IM injections of tramadol (5 mg/kg, Alborz Darou, Iran) administered half an hour before the surgery as well as every 12 hours for two days following surgery.

### Preparation of tissue sections for histological observation

Animals were deeply anesthetized by the use of 10% ketamine (Parke-Davis, Detroit, MI, USA) and subsequently sacrificed by an overdose of anesthetic drug. The jaws were cut and samples placed in 10% formalin, after which samples were placed in 10% nitric acid for 14 days in order to become decalcified. After decalcification, samples were again placed in 10% formalin for 24 hours and subsequently dehydrated by ascending concentrations of an alcohol solution, as follows. Samples were first immersed for 1.5 hours in 70% alcohol, 1.5 hours in 80% alcohol, 2.5 hours in 96% alcohol and 2.5 hours in 100% alcohol. This was followed immersion for 2 hours in xylol and 8 to 18 hours in melted paraffin at a temperature of 56˚C to 67˚C.

### Sectioning technique and paraffin/histology protocol

Paraffin embedded samples were cut by a rotary microtome to provide slides. Each sample provided multiple mesio-distal slides of 5 μm thicknesses each. Slides were placed for 30 minutes in an oven at 80˚C-110˚C and were subsequently stained with hematoxylin and eosin (H&E).

### Evaluation of RR

There were 16 H&E stained sections used to determine RR scores according to the magnified photographs of apical RR. Adobe Photoshop® software was used to measure bone histomorphometric parameters. A grid-sheet used for the preceding evaluation was superimposed in the same way and the numbers of grids with or without resorption lacuna were measured separately. RR scores as the percentage of resorption grids were determined by dividing the numbers of grids with resorption lacuna by the total numbers of grids along the root surface.

### Evaluation of bone resorption, angiogenesis, osteogenesis and inflammation around teeth

Following preparation of the slides, histomorphometric analysis was performed for bone resorption, angiogenesis, osteogenesis and eventual inflammation around teeth following microscopic evaluations.

### Statistical analysis

Means and standard deviations were calculated for each group. Univariante 3-way ANOVA test was used to evaluate the effects of intervention, different experimental periods and jaws on tooth movement and RR. Statistical package for the social sciences (SPSS) software was used and the significance level set at p<0.05.

## Results

### OTM

Table 1 illustrates the values obtained for OTM in the experimental and control groups with an orthodontic appliance. All measurements during the stages of tooth movement are shown. NanoBone® reduced the amount of tooth movement.

According to the findings, a trend in tooth movement reduction was seen from first month to the second month 1.205 ± 0.144 mm to 0.856 ± 0.154 mm in the experimental groups respectively.

In the mandible, tooth movement was 1.035 ± 0.075 mm compared to 1.117 ± 0.165 mm for the maxilla. There was no significant difference between the groups (p>0.05).

**Table 1:** Mean ± standard deviation for tooth movement (mm) at each phase according to group and jaw


Force duration	OTM (mm)	Experimental group (mm)	Control group (mm)	Number

**1 month**	Maxilla	1.425 ± 0.185	1.402± 0.157	4
	Mandible	0.985 ± 0.104	1.036± 0.104	4
	Total	1.205 ± 0.144	1.219± 0.130	8
**2 months**	Maxilla	0.911 ± 0.204	1.109± 0.0307	4
	Mandible	0.802 ± 0.104	1.005± 0.0707	4
	Total	0.856 ± 0.154	1.057± 0.050	8
**Total**	Maxilla	1.117 ± 0.165	1.255± 0.203	8
	Mandible	1.035 ± 0.075	1.150± 0.107	8


OTM; Orthodontic tooth movment.

Table 2 illustrates the values obtained for RR in the experimental and control groups. No experimental groups exhibited any significant scores when compared with the corresponding controls. The values were lower in the two-month group than in the onemonth group (22.92 ± 11.685 mm^2^ in the first stage of measurement compared with 21.3565 ± 11.17 mm^2^ in the second stage). In the mandible this measurement was less prominent than the maxilla (21.406 ± 11.62 mm^2^ compared to 22.87 ± 11.25 mm^2^). We observed no significant differences among the two experimental groups with different durations of the force application and the upper or lower jaw (p>0.05).

### Evaluation of bone resorption, angiogenesis, osteogenesis and inflammation surrounding the teeth

**Fig 5:**
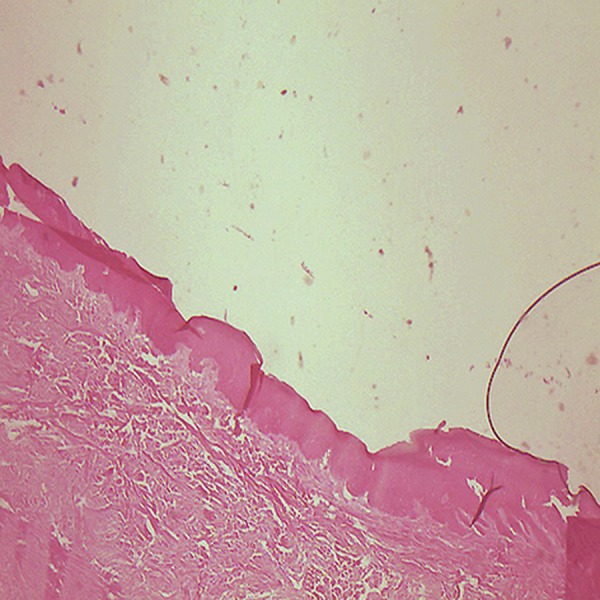
Para-keratinized epithelium covered the mouth mucosa, (magnification ×10).

**Fig 6:**
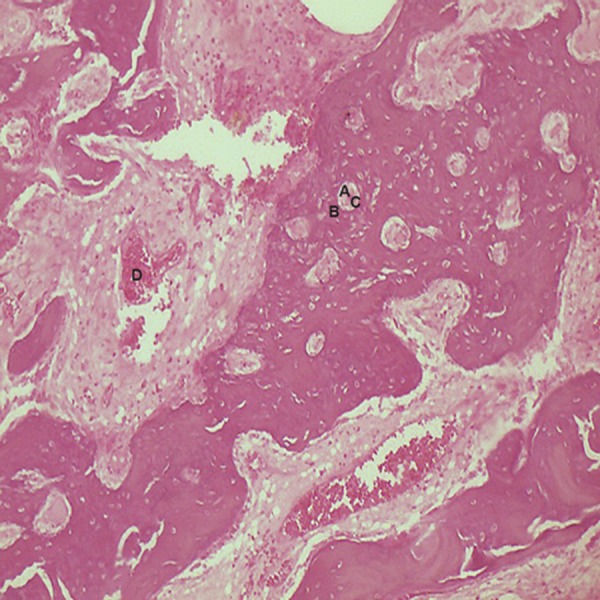
Angiogenesis-small and large endothelium-lined channels that are engorged with red blood cells. Note: New bone formation around NanoBone® with osteocytic lacuna and an osteoblastic rim(magnification ×10). A; NanoBone® remnants, B; Osteoblastic rim, C; Osteocytic lacuna and D; Angiogenesis.

**Fig 7:**
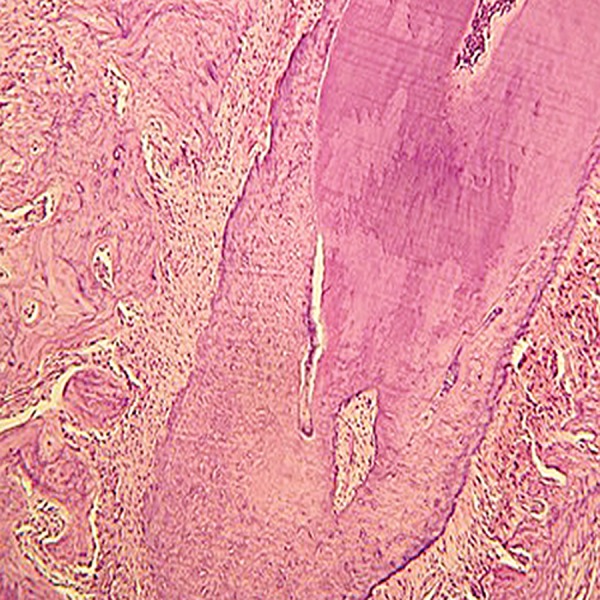
No significant root resorption observed. Note the thick secondary cellular cementum (magnification ×40).

During the histopathological evaluation of the left canines and first premolars of both upper and lower jaws, we observed a wide lining of para-keratinized epithelium that covered the mouth mucosa (Fig 5). The underlying connective tissue showed collagen fibers and mild infiltration of chronic inflammatory cells as well as traces of new capillary formation and angiogenesis (Fig 6). Bone trabeculae with osteoblastic rim accompanied by active clusters of osteoblasts and lacunas that contained osteocytes were observed around the NanoBone® remnants (Fig 6). Around the above mentioned teeth, traces of osteogenesis, a thick layer of second cellular cementum and minimal evidence of surface and circumferential RR were present (Fig 7).

**Fig 8:**
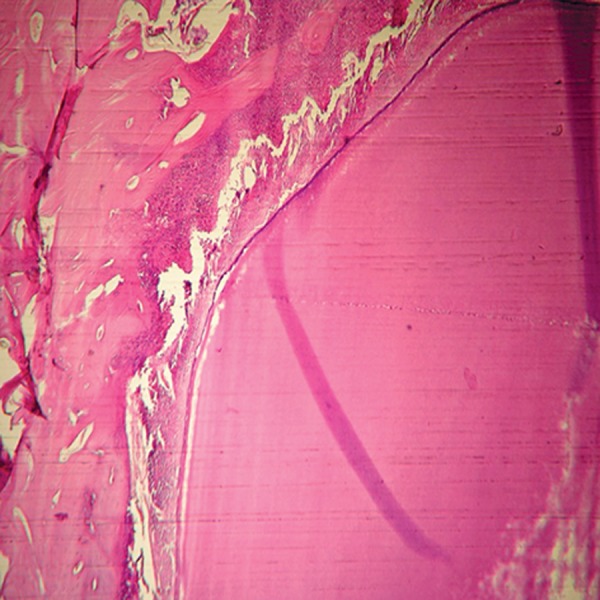
Osteoblastic rim and osteocytes in the control group. Note the primary cementum (magnification ×40).

In the histopathological assessment of samples from the right canines and first premolars of the upper and lower jaws of the control group, we observed a layer of para-keratanized epithelium which covered the mucosa of the mouth. In the underlying connective tissue, collagen fibers and a mild infiltration of chronic inflammatory cells were seen. The bone around the teeth (control group) was normal with no traces of angiogenesis, osteogenesis, orRR around the teeth (Fig 8).

The number of resorptive lacunae were more in the maxillary jaw relative to the lower jaw (Figes9, 10). This number decreased after eight weeks in both jaws relative to the control group (Fig 11).

**Table 2:** Root resorption (RR) scores in the experimental and control groups


Force duration	RR (×10^-4^ mm^2^)	Experimental group (×10^-4^ mm^2^)	Control group(×10^-4^ mm^2^)	Number

**1 month**	Maxilla	23.83± 12.27	22.402 ± 13.17	4
	Mandible	22.01± 11.10	21.03± 12.14	4
	Total	22.92± 11.685	21.716 ± 12.655	8
**2 months**	Maxilla	21.911 ± 10.20	19.19± 8.03	4
	Mandible	20.802 ± 12.14	19.23± 11.07	4
	Total	21.3565 ± 11.17	19.21± 9.55	8
**Total**	Maxilla	22.87± 11.25	20.79± 1058	8
	Mandible	21.406 ± 11.62	20.13± 11.605	8


RR; Root resorption.

**Fig 9:**
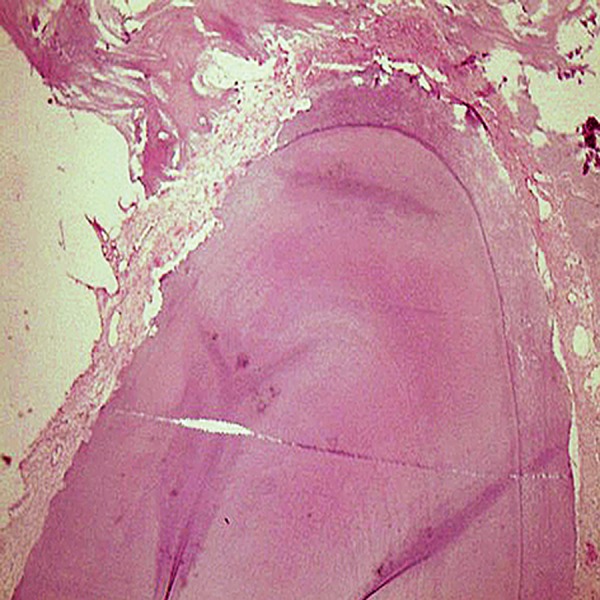
Resorption lacuna in the upper jaw teeth after one month (magnification ×40).

**Fig 10:**
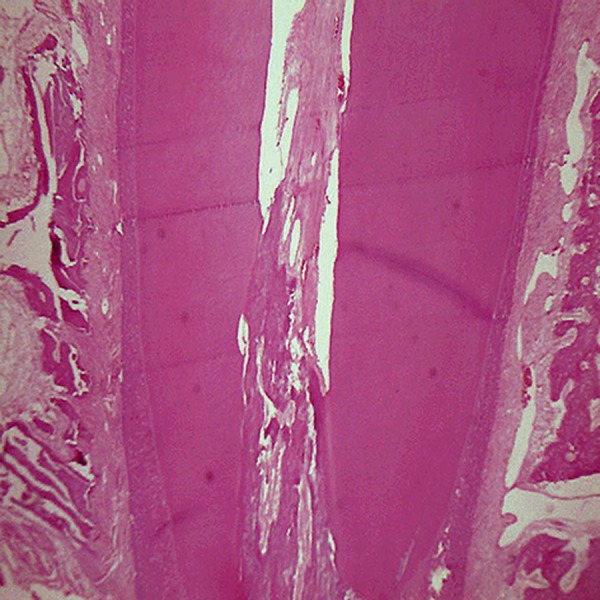
Resorption lacuna in the lower jaw teeth after one month (magnification ×40).

**Fig 11:**
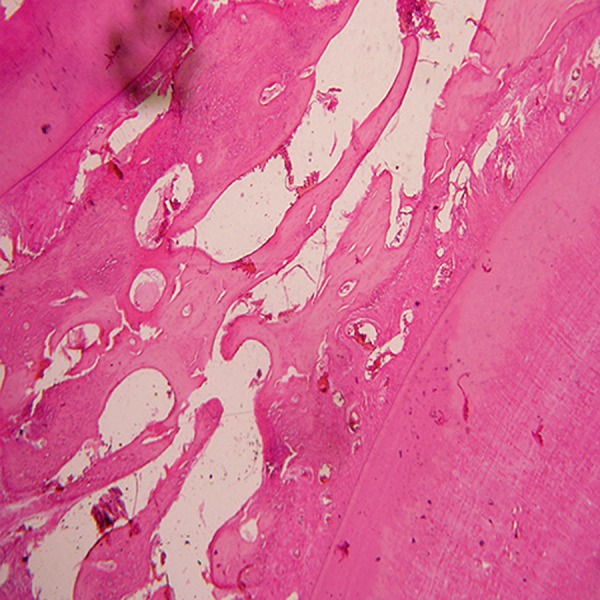
The number of resorption lacuna decreased in two months (magnification ×40).

## Discussion

The use of NanoBone® in the socket of an extracted tooth can preserve the alveolar bone and the adjacent teeth can be moved through the extraction site by an orthodontic force without significant RR. Inspite of alveolar bone preservation, no significant difference was observed between control and experimental groups in terms of OTM. Orthodontic socket preservation enhanced the health condition of the periodontium located in proximity to the extraction site.

Seifi et al. (10) evaluated the effects of demineralized freeze-dried bone allograft (DFDBA) and Freeze-Dried Bone Allograft (FDBA) applicationson OTM and histologically assessed the results. They reported that DFDBA and FDBA graft materials could be used as autogenic bone replacement in order to move teeth in jaw defects or extraction sites. The above mentioned study showed that after the application of an orthodontic force, teeth from both groups moved mesially at the rate of 1.2 mm per month which was similar to our study and agreed with the findings reported by Araujo et al. (40) who reported a rate of tooth movement of approximately 1 mm/month which was also similar to the current study. Hossain et al. (41) reporteda movement of 2 mm/month in tricalcium phosphate ceramics. This rate might be attributed to the fact that they moved a central incisor.

The present research showed that synthetic bone substitute enhanced angiogenesis and osteogenesis in the experimental group compared to the control group. The data corroborated the findings of Henkel et al. (35), Gotz et al. (30) and Schwarz et al. (42).

Henkel et al. (35) studied the inductive characteristics of bone formation and biodegradation of different materials with a calcium phosphate basis. Histologic, morphologic and macroscopic evaluations of areas of previous lesions after ten months showed that calcium-based material resulted in complete bone formation in regions of repaired lesions and the foreign material was almost completely absorbed. Yet after disposition of previously used materials bone formation was insufficient and the amount of absorption of the foreign substance was considered weak. In this research they concluded that bone formation induction properties of calcium phosphate-based material was better compared to previously used materials, the safety of this material for repair of bone lesions was appropriate, and it had a particular importance for dentists such as implant surgeons and orthopedists.

Gotz et al. (30) assessed the immunohistochemical properties of hydroxyapatite nanocrystalline silica gel on biopsies obtained from human jaw bones. Based on the results of the study, they concluded that NanoBone® had osteoconductive and biomimetic properties and was integrated into the host’s physiological bone turnover at a very early stage.

Schwarz et al. (42) investigated the treatment results of peri-implant lesions following application of NHA. Tissue analysis demonstrated high absorption and differentiation of NHA and osteoconductive material as well as absorption of other substances. In addition, it had a major role in preservation of the alveolar ridge after tooth extraction and could have positive effects on improvement of wounds and prevention of bone atrophy.

Gotz et al. (43) assessed the probable osteoinductive properties of NanoBone®. Granules were implanted subcutaneously and intramuscularly into the back regions of 18 mini-pigs. After periods of five weeks, ten weeks, four months and eight months, they investigated the implantation sites using histological and histomorphometric procedures. Signs of early osteogenesis were detected after five weeks. The later periods were characterized by increasing membranous osteogenesis in and around the granules that led to the formation of bone-like structures which showed periosteal and tendon-like structures with bone marrow and focalchondrogenesis. Thus, the results of this preliminary study indicated that this biomaterial has osteoinductive potential and induced the formation of bone structures. As a basic phenomenon in NanoBone®, substitution of the original SiO_2_ gel matrix by organic molecules formed an organic matrix around the embedded hydroxyapatite which seemed to be the key event that caused these results (30, 34). Although no specific tissue reaction could be related to the described silica degradation, the biomaterial was close to being fully degraded without a severe inflammatory response. These characteristics were advantageous for bone regeneration and remodeling processes (28).

The mean RR in the synthetic bone substitute group was 22.87 ± 11.25 ×10^-4^ mm^2^ in the maxilla and 21.406 ± 11.62 ×10^-4^ mm^2^ in the mandible. ANOVA analysis did not show any significant difference compared to the control group (19.19 ± 8.03 ×10^-4^ mm^2^ in the maxilla and 20.13 ± 11.605 ×10^-4^ mm^2^ in the mandible, p>0.05) which agreed with the findings of Kasaj et al. (44) who assessed the ability of NHA paste to promote human periodontal ligament cell proliferation. The findings of their study indicated that NHA was a stimulator of cell proliferation and the mitogenic effect of NHA paste was mediated by epidermal growth factor receptor (EGFR). Since PDL contains several cell populations that include fibroblasts, cementoblasts, osteoblastic and osteoclastic cells, and mesenchymal cells (45) thus activation of cells derived from PDL play an important role in periodontal regeneration. This might have led to decreased RR in the present study.

## Conclusion

Nanocrystalline hydroxyapatite as a synthetic bone substitute can preserve the alveolar socket of extracted teeth for orthodontic purposes (orthodontic socket preservation) and will not interfere with OTM. The use of synthetic bone substitute can induce neovascularization and osteogenesis, and it does not have a major impact on the amount of RR. Further studies are required for determination of the role of numerous intervening factors.
